# HU-based material conversion for BNCT accurate dose estimation

**DOI:** 10.1038/s41598-023-42508-0

**Published:** 2023-09-21

**Authors:** Yi-Chiao Teng, Jiang Chen, Wan-Bing Zhong, Yuan-Hao Liu

**Affiliations:** 1 Neuboron Therapy System Ltd., Xiamen, Fujian People’s Republic of China; 2 National Tsing Hua University, Hsinchu, 30013 Taiwan Republic of China; 3 Nanjing Vocational University of Industry Technology, Nanjing, Jiangsu People’s Republic of China; 4 Nanjing University of Aeronautics and Astronautics, Nanjing, Jiangsu People’s Republic of China; 5 Neuboron Medtech Ltd., Nanjing, Jiangsu People’s Republic of China; 6 Xiamen Humanity Hospital, Xiamen, Fujian People’s Republic of China

**Keywords:** Nuclear physics, Materials science, Physics, Medical imaging

## Abstract

NeuMANTA is a new generation boron neutron capture therapy (BNCT)-specific treatment planning system developed by the Neuboron Medical Group and upgraded to an important feature, a Hounsfield unit (HU)-based material conversion algorithm. The range of HU values was refined to 96 specific groups and established corresponding to tissue information. The elemental compositions and mass densities have an important effect on the calculated dose distribution. The region of interest defined in the treatment plan can be converted into multiple material compositions based on HU values or assigned specified single material composition in NeuMANTA. Different material compositions may cause normal tissue maximum dose rates to differ by more than 10% in biologically equivalent doses and to differ by up to 6% in physically absorbed doses. Although the tumor has a lower proportion of BNCT background dose, the material composition difference may affect the minimum dose of biologically equivalent dose and physically absorbed dose by more than 3%. In addition, the difference in material composition could lead to a change in neutron moderation as well as scattering. Therefore, the material composition has a significant impact on the assessment of normal tissue side effects and tumor control probability. It is essential for accurate dose estimation in BNCT.

## Introduction

**Figure 1:**
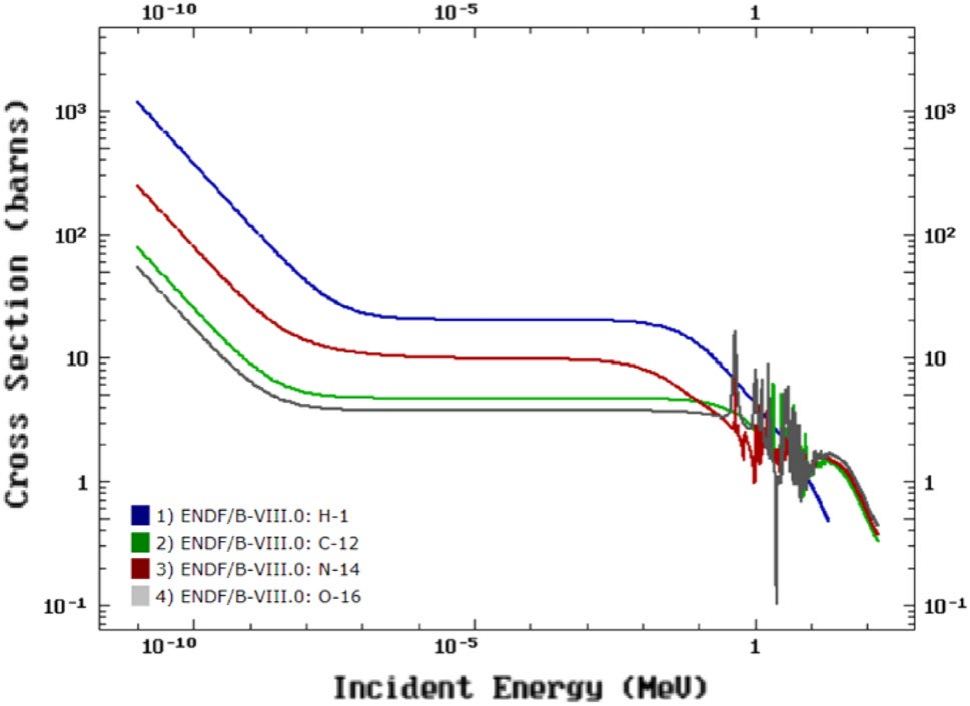
Neutron cross-section of main tissue elements from ENDF/B-VIII.0

The physically absorbed dose of boron neutron capture therapy (BNCT) consists of boron dose, neutron dose, and photon doses, where boron dose is generated by the ^10^B(n,α)^7^Li reaction, neutron dose is mainly generated by the ^1^H(n,n′)p and ^14^N(n,p)^14^C reactions, and photon dose is induced by the primary photon from the beam exit and the secondary photon from ^1^H(n,γ)^2^H, ^16^O(n,γ)^17^O and ^12^C(n,γ)^13^C reactions^1,2^. The background dose of BNCT includes neutron and photon doses, which are generated mainly by the interaction of the elements of human tissue with neutrons. Neutron cross-sections of the main tissue elements retrieved from ENDF/B-VIII.0 are shown in Fig. 1^3^. As one can tell, every nuclide has its own unique neutron cross-section. Therefore, the elemental composition of tissue material has an important influence on dose calculation because of the difference in interactions of neutrons, and the correct composition of tissue material directly affects the final correctness of the dose calculation.

The elemental composition of the detailed voxel model used in Monte Carlo (MC) dose engine is an important factor affecting the correctness of the dose calculation. In radiotherapy, computed tomography (CT) images are displayed by using a Hounsfield Unit (HU, unitless). The HU value can be applied to convert the physical density, electron density, or stopping power of the material for the calculation and evaluation of dose distribution^4,5^. However, most BNCT treatment planning systems (TPSs) calculate the dose with the average tissue composition described in the ICRU-46 report^6^ rather than obtaining material information directly from CT images.

Currently, most TPSs for BNCT, such as SERA, Tsukuba Plan, and THORplan^7–9^, describe different regions of interest (ROIs) with heterogeneous material composition but define the voxel material in ROIs (i.e., interROI heterogeneous but intraROI homogeneous model) with single and homogeneous material composition. However, NeuMANTA^10^ (Multifunctional Arithmetic for Neutron Transportation Analysis) is a new generation BNCT-specific TPS developed by Neuboron Medical Group. NeuMANTA pioneered the application of heterogeneous element composition to define the voxel material in ROIs (i.e., interROI and intraROI heterogeneous model), which can effectively improve the accuracy of the voxel model. In this regard, NeuMANTA has proposed a method to obtain a more realistic material composition from the HU value of CT images.

## Methods

To obtain the material elemental composition and density of each voxel more precisely, NeuMANTA introduces a method to define the material information of a voxel as per the HU values of CT. The grayscale value of CT is expressed in Hounsfield Units (a.k.a. Hounsfield Scale), which are defined as:

1 $$HU=1000\times \frac{{\mu }_{tissue}-{\mu }_{water}}{{\mu }_{water}-{\mu }_{air}}$$

where ${\mu }_{tissue}$, ${\mu }_{water}$, and ${\mu }_{air}$ are the linear attenuation coefficients for tissue, water, and air, respectively^11^. Water is defined as HU = 0 and air as HU =  − 1000. The effective linear attenuation coefficient of the tissue can be obtained as per the average linear attenuation coefficient ${\mu }_{i}$ of the basic elements and the mass ratio of tissue element ${m}_{i}$ in the CT energy range provided by the National Institute of Standards and Technology (NIST):

2 $${\mu }_{tissue}=\sum {m}_{i}{\mu }_{i}$$

**Figure 2:**
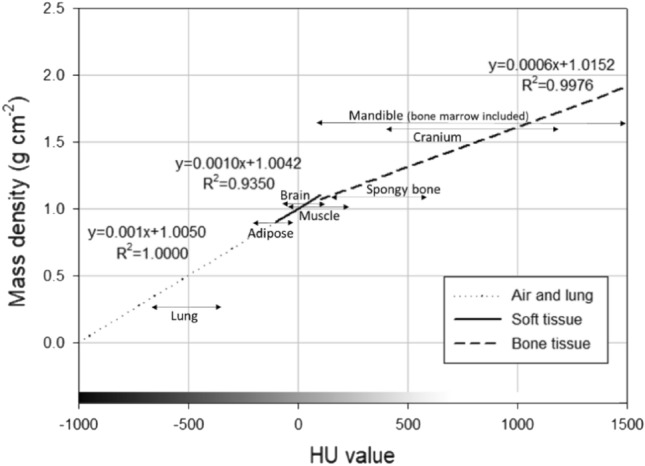
Relationship between HU value and mass density.

According to the studies of Fang^12^, Sudhyadhom^13^, and Schneider^14^, the elemental composition and density of soft and skeletal tissues in CT images can be obtained based on the conversion of HU values; the relationship between HU value (x) and mass density (y) is shown in Fig. 2:

The relationship between the HU value and mass density in the range of HU values below -100 for air (sinuses and trachea) and lung tissue, in the range between -100 and 100 for soft tissue, and the HU value above 100 for skeletal tissue was used as follows^15^:

3 $$y=0.0010x+1.0050\, \mathrm{for\, air\, and\, lung\, }(\mathrm{HU}<-100)$$

4 $$y=0.0010x+1.0042\, \mathrm{for\, soft\, tissue\, }(-100<\mathrm{HU}<100)$$

5 $$y=0.0006x+1.0152\, \mathrm{for\, skeletal\, tissue\, }(\mathrm{HU}>100)$$

Due to the small number of references defining the elemental composition and density of HU-based conversion, some HU values have a large range, which makes it difficult to identify the differences in tissue density. Therefore, NeuMANTA refined the range of HU values, grouping HU values below − 950 with an interval of 10 HUs, HU values within − 950 to − 100 and 100 to 1,00 with an interval of 25 HUs, HU values within − 100 to 100 with an interval of 20 HUs, and HU values greater than 1000 with an interval of 50 HUs. In total, 96 specific HU value groups were established corresponding to tissue information^16^, as shown in Appendix A. The tissue material database can be applied to soft and skeletal tissues such as adipose tissue, muscle, brain tissue, lymph, bone marrow, cranium, cortical bone, and mandible. The ROI defined in the treatment plan can then be converted into multiple material compositions based on HU values.

However, there are limitations to using this conversion method. The CT images applied for treatment plans are limited by spatial resolution or slice thickness, and therefore, some of the organs at risk (OARs) may not be clearly presented on CT images, such as skin and oral mucosa. As a result, the material information for the OARs retrieved from HU values may be less accurate or incorrect. In this case, to avoid acute side effects caused by normal tissue receiving more than the maximum tolerance dose during BNCT, users can perform a conservative dose evaluation in NeuMANTA by assigning a specified single material composition.

Users of NeuMANTA can define the ROI as heterogeneous material compositions or a single homogeneous material via the built-in tissue material database. Once the user completes the settings, NeuMANTA creates a 3D encoding matrix with elemental composition and density through medical images based on the user settings to generate the detailed voxel model required for MC calculations^17,18^. Because of the individual difference of patients in geometry and body tissue distribution, the 3D model created by NeuMANTA is expected to be more realistic, and the dose calculation results are more trustworthy than a treatment plan using single material composition in defining ROIs.

To investigate the difference between both modalities (intraROI homogeneous model versus intraROI heterogeneous model), we analyzed CT images of brain tumor case provided by Peking Union Medical College Hospital and head-and-neck cancer (HNC) case provided by Xiamen Humanity Hospital with an X-ray tube voltage of 120 kVp. A single image is 512 × 512 pixels. The voxel size was 0.68 × 0.68 × 2.5 mm^3^ for the brain tumor case and 1.17 × 1.17 × 2.5 mm^3^ for the HNC case. The calculated ROI doses using the two modalities are compared and analyzed.

The images provided by Peking Union Medical College Hospital were approved by the Institutional Review Board (IRB) under the National Key Research and Development Program (Grant No. 2017YFC0107700); the images provided by Xiamen Humanity Hospital were also approved by its IRB under the Science and Technology Major Project of the Xiamen Municipal Bureau of Science and Technology (Grant No. 3502720201031). Informed consent was obtained from all subjects or their legal guardians and all methods were carried out in accordance with relevant guidelines and regulations.

In both the brain and HNC cases, boron concentration within normal tissue was identified at 25 parts per million (ppm), while within tumorous tissue it increased to 62.5 ppm. The physically absorbed dose of BNCT is described in Eq. (6), where D_B_, D_N,_ and D_P_ respectively denote the physically absorbed doses of boron, neutron, and photon, with all doses presented in Gray (Gy). The biologically equivalent dose, incorporated in Eq. (7), considers weighted factors such as Relative Biological Effectiveness (RBE) and Compound Biological Effectiveness (CBE). The units are designated in Gray Equivalent (Gy-Eq). The values incorporated in this research are as follows: RBE = 3.2 for neutrons and RBE = 1.0 for photons; CBE = 2.5 for skin, CBE = 1.3 for other normal tissues, and CBE = 3.8 for tumors.

6 $${D}_{total,phy} (Gy)={D}_{B}+{D}_{N}+{D}_{P}$$

7 $$D_{total,BE} \left( {Gy - Eq} \right) = D_{B} \cdot CBE + D_{N} \cdot RBE_{N} + D_{P} \cdot RBE_{P}$$

In this investigation, an accelerator-based BNCT system, NeuPex, in conjunction with the Monte Carlo dose engine, COMPASS^16^ (COMpact PArticle Simulation System), was deployed for the computation of dose distribution. The NeuPex system employs a stationary lithium target and a proton beam with a nominal energy of 2.5 MeV and a proton current of 10 mA. The COMPASS simulation processed 1.0E + 9 particle histories, executing on an Intel(R) Core(TM) i9-7940X processor. The computation duration for two different conversion models—intraROI homogeneous model and intraROI heterogeneous model—was evaluated based on a single CPU core. For the brain case involving 8.1 million cells, the approximate calculation time was 110 min. Conversely, for the HNC scenario, which comprised 2.7 million cells, the computation was notably quicker, approximately taking 55 min.

## Results

**Figure 3:**
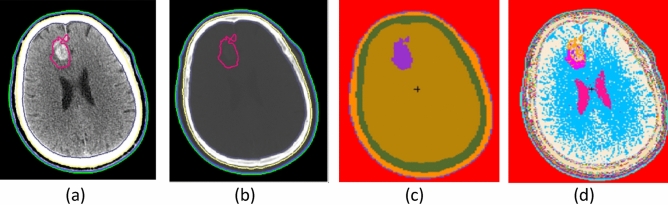
Brain tumor case: (**a**) CT image in the brain window. (**b**) CT image in the bone window. (**c**) Conversion model I with specific material from the ICRU-46 report in ROIs. (**d**) Conversion model II with multiple materials that were automatically converted to the elemental compositions and densities by the HU values.

For the sake of emphasizing the details of brain and skeletal tissues in CT images of the brain tumor cases, Fig. 3a and b are shown using the windowing method. Figure 3a is shown using brain windows (WW: 80, WL: 40), while Fig. 3(b) is shown using bone windows (WW: 2800, WL: 600)^19^. The ROI-GTV (gross tumor volume) of the red line in Fig. 3a has areas in which the density is obviously different from brain tissue density; the ROI-brain (gray) shows gray matter, white matter, and cerebrospinal fluid. The ROI-skeletal tissue (yellow) in Fig. 3b shows different gray values, indicating heterogeneous skull bone. The conversion model I in Fig. 3c is provided with the average tissue material composition for ROIs as suggested by the International Commission on Radiological Units and Measurements (ICRU) (i.e., interROI heterogeneous and intraROI homogeneous model). The conversion model II in Fig. 3d is provided with the skin tissue material composition for ROI-skin as suggested by ICRU, and the rest of the ROIs are provided with predefined materials whose elemental compositions and densities are described in Appendix A converted by the HU of images (i.e., inter- and intraROI heterogeneous model). The difference in material distribution between the two material determination methods can be clearly seen in Fig. 3c and d; the butterfly-shaped ventricle of the brain can be clearly seen in Fig. 3d, while this area is not visible in Fig. 3c.

The HU of GTV in the transformed voxel model has a range of 24–170, with the brain density of 1.04 g/cm^3^ and composition of ICRU-46 adopted in model I and the density of 1.3043–1.3480 g/cm^3^ transformed from HU values adopted in model II. The HU of normal brain tissue has a range of 3–88, and the brain composition in ICRU is adopted in model I, while the density of 1.29–1.3257 g/cm^3^ is adopted in model II. The HU of soft tissue ranges from − 46 to 81, with a density of 1.00 g/cm^3^ adopted in model I and 1.2671–1.3257 g/cm^3^ adopted in model II. The HU of skeletal tissue ranges from − 965 to 2048, with a density of 1.61 g/cm^3^ adopted in model I and 0.97–1.92 g/cm^3^ adopted in model II.

**Figure 4:**
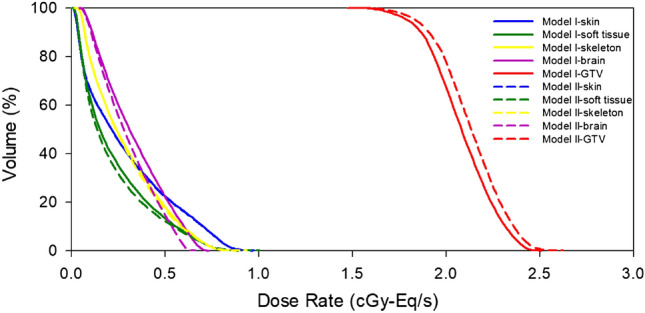
DVHs of brain tumor case of conversion models I and II.

Table 1 shows the results of the biologically equivalent dose rate, the mean statistical error of the ROI, and the difference between conversion models I and II. The number of particle histories in the MCNP simulation is 1.0E + 9. The calculation error between the mean dose rate (${\dot{D}}_{mean}$) and maximum dose rate (${\dot{D}}_{max}$) is shown in parentheses; the statistical error of the minimum dose rate (${\dot{D}}_{min}$) for other ROIs, except for the tumor, is not meaningful; therefore, they are not listed in the table. The calculation error of ${\dot{D}}_{min}$ for GTV is 0.93% versus 0.94% for model I and model II, respectively. Except for skin tissue, there were significant differences in ${\dot{D}}_{max}$ for all normal tissues. Generally, in brain tumor clinical cases, it is important to pay attention to the mean and maximum doses in the normal brain and ensure that they are tolerable. The difference between models I and II was more than 10%, which indicates a significant effect of different material compositions on dose evaluation. The difference in the maximum tumor dose rate between both models reached 5.2%, and the difference in the mean dose rate reached 2.3%. Differences of several percentage points are found in all other tissues. The biologically equivalent dose distribution of each ROI was also found to be different between the two models, as shown in Fig. 4. From these results, the element composition and density of tissue material clearly have a high impact on dose distribution, and the application of single homogeneous material composition for ROI material definition does not consider the individual variation and may result in distorted dose evaluation.

**Table 1:** Biologically equivalent dose rate results of brain tumor case of conversion models I and II.

ROI	Conversion model I (IntraROI homogeneous)				Conversion model II (IntraROI heterogeneous)				Difference		
	Mat	${\dot{D}}_{max}$(cGy-Eq/s)	${\dot{D}}_{min}$(cGy-Eq/s)	${\dot{D}}_{mean}$(cGy-Eq/s)	Mat	${\dot{D}}_{max}$(cGy-Eq/s)	${\dot{D}}_{min}$(cGy-Eq/s)	${\dot{D}}_{mean}$(cGy-Eq/s)	${\dot{D}}_{max}$	${\dot{D}}_{min}$	${\dot{D}}_{mean}$
Skin	ICRU_Skin_	0.962 (1.04%)	0.013	0.290 (2.66%)	ICRU_Skin_	0.982 (1.11%)	0.013	0.289 (2.64%)	2.1%	1.2%	-0.2%
Soft Tissue	ICRU_ST_	0.885 (1.25%)	0.013	0.228 (3.03%)	HU conv	1.004 (1.16%)	0.012	0.216 (3.07%)	13.5%	− 10.0%	− 5.4%
Skeletal Tissue	ICRU_Cranium_	0.872 (1.13%)	0.022	0.290 (2.27%)	HU conv	0.934 (1.07%)	0.022	0.285 (2.27%)	7.2%	3.8%	− 1.8%
Brain	ICRU_Brain_	0.730 (1.03%)	0.047	0.333 (1.68%)	HU conv	0.648 (1.10%)	0.046	0.300 (1.75%)	− 11.3%	− 0.4%	− 10.0%
GTV	ICRU_Brain_	2.495 (0.69%)	1.495	2.083 (0.75%)	HU conv	2.624 (0.67%)	1.490	2.132 (0.74%)	5.2%	− 0.3%	2.3%

Mat.: Material, ST: Soft Tissue, HU conv.: HU conversion.

Further analysis of the dose component was performed to explore the effect of different element compositions and densities on the dose. Table 2 shows the biologically equivalent dose component of the maximum normal tissue dose rate, maximum GTV dose rate, and minimum GTV dose rate, and the proportions in parentheses. The total dose rate is the sum of the boron dose rate ($\dot{{D}_{B}}$), neutron dose rate ($\dot{{D}_{N}}$), and photon dose rate ($\dot{{D}_{P}}$). The skin of both models uses the same reference element composition and density from ICRU, but the proportions of biologically equivalent dose components differ significantly, affected by neutron backscattering due to the different soft tissue and skeletal tissue of the two models. The background dose rate ($\dot{{D}_{N}}$ + $\dot{{D}_{P}}$) of soft tissue, skeletal tissue, and brain accounts for approximately 41 to 56% of the maximum total biologically equivalent dose rate, while the background dose rate of GTV accounts for less than 19% of the maximum and minimum biologically total equivalent dose rate in both models.

**Table 2:** Biologically equivalent dose rate component of brain tumor case of conversion models I and II.

ROI	Dose category	Conversion model I (IntraROI homogeneous)			Conversion model II (IntraROI heterogeneous)			Difference		
		${\dot{D}}_{B}$(cGy-Eq/s)	${\dot{D}}_{N}$(cGy-Eq/s)	${\dot{D}}_{P}$(cGy-Eq/s)	${\dot{D}}_{B}$(cGy-Eq/s)	${\dot{D}}_{N}$(cGy-Eq/s)	${\dot{D}}_{P}$(cGy-Eq/s)	${\dot{D}}_{B}$	${\dot{D}}_{N}$	${\dot{D}}_{P}$
Skin	${\dot{D}}_{max}$	0.448 (46.6%)	0.372 (38.6%)	0.142 (14.8%)	0.739 (75.3%)	0.118 (12.0%)	0.125 (12.7%)	64.7%	− 68.3%	− 12.1%
Soft tissue	${\dot{D}}_{max}$	0.526 (59.4%)	0.176 (19.9%)	0.183 (20.7%)	0.552 (54.9%)	0.287 (28.6%)	0.166 (16.5%)	5.0%	63.1%	− 9.4%
Skeletal tissue	${\dot{D}}_{max}$	0.478 (54.9%)	0.189 (21.7%)	0.204 (23.4%)	0.463 (49.6%)	0.290 (31.0%)	0.181 (19.4%)	− 3.2%	53.0%	− 11.2%
Brain	${\dot{D}}_{max}$	0.323 (44.2%)	0.160 (22.0%)	0.247 (33.8%)	0.327 (50.5%)	0.100 (15.4%)	0.220 (34.0%)	1.2%	− 37.7%	− 10.7%
GTV	${\dot{D}}_{max}$	2.135 (85.6%)	0.125 (5.0%)	0.236 (9.4%)	2.125 (81.0%)	0.305 (11.6%)	0.193 (7.4%)	− 0.5%	144.4%	− 18.0%
	${\dot{D}}_{min}$	1.241 (83.7%)	0.096 (6.5%)	0.146 (9.9%)	1.297 (87.0%)	0.064 (4.3%)	0.130 (8.7%)	4.5%	− 33.4%	− 10.8%

Table 3 shows the results of the physically absorbed dose rate and the differences between conversion model I and II. The difference between the two models is up to 7.2% in maximum brain physically absorbed dose rate and 6.1% in mean dose rate. The difference in minimum GTV dose rate was 2.2%. These differences indicate a significant effect of different material compositions on dose evaluation. Table 4 shows the physically absorbed dose component and the proportions in parentheses. The background dose rate of normal tissue accounts for up to 55% of the maximum total physically absorbed dose rate, while the background dose rate of GTV accounts for less than 35% of the maximum and minimum total physically absorbed dose rates.

**Table 3:** Physically absorbed dose rate results of brain tumor case of conversion models I and II.

ROI	Conversion model I (IntraROI homogeneous)				Conversion model II (IntraROI heterogeneous)				Difference		
	Mat	${\dot{D}}_{max}$(cGy/s)	${\dot{D}}_{min}$(cGy/s)	${\dot{D}}_{mean}$(cGy/s)	Mat	${\dot{D}}_{max}$(cGy/s)	${\dot{D}}_{min}$(cGy/s)	${\dot{D}}_{mean}$(cGy/s)	${\dot{D}}_{max}$	${\dot{D}}_{min}$	${\dot{D}}_{mean}$
Skin	ICRU_Skin_	0.453 (0.84%)	0.013	0.141 (2.68%)	ICRU_Skin_	0.463 (1.03%)	0.012	0.138 (2.64%)	2.3%	− 6.1%	− 2.6%
Soft tissue	ICRU_ST_	0.642 (0.87%)	0.013	0.165 (2.52%)	HU conv	0.682 (0.82%)	< 0.001	0.150 (2.53%)	6.2%	–	− 9.0%
Skeletal tissue	ICRU_Cranium_	0.630 (0.84%)	0.021	0.213 (1.88%)	HU conv	0.636 (0.82%)	< 0.001	0.207 (1.83%)	0.9%	–	− 2.7%
Brain	ICRU_Brain_	0.551 (0.94%)	0.044	0.259 (1.60%)	HU conv	0.512 (0.93%)	0.043	0.244 (1.58%)	− 7.2%	− 3.3%	− 6.1%
GTV	ICRU_Brain_	0.837 (0.78%)	0.503	0.704 (0.84%)	HU conv	0.848 (0.73%)	0.492	0.699 (0.82%)	1.3%	− 2.2%	− 0.7%

Mat.: Material, ST: Soft Tissue, HU conv.: HU conversion.

**Table 4:** Physically absorbed dose rate component of brain tumor case of conversion models I and II.

ROI	Dose Category	Conversion model I (IntraROI homogeneous)			Conversion model II (IntraROI heterogeneous)			Difference		
		${\dot{D}}_{B}$(cGy/s)	${\dot{D}}_{N}$(cGy/s)	${\dot{D}}_{P}$(cGy/s)	${\dot{D}}_{B}$(cGy/s)	${\dot{D}}_{N}$(cGy/s)	${\dot{D}}_{P}$(cGy/s)	${\dot{D}}_{B}$	${\dot{D}}_{N}$	${\dot{D}}_{P}$
Skin	${\dot{D}}_{max}$	0.279 (61.6%)	0.032 (7.1%)	0.142 (31.3%)	0.296 (63.9%)	0.034 (7.2%)	0.133 (28.8%)	6.1%	4.6%	− 6.1%
Soft tissue	${\dot{D}}_{max}$	0.404 (63.0%)	0.055 (8.6%)	0.183 (28.5%)	0.427 (62.6%)	0.087 (12.8%)	0.168 (24.6%)	5.6%	58.4%	− 8.0%
Skeletal tissue	${\dot{D}}_{max}$	0.368 (58.3%)	0.059 (9.4%)	0.204 (32.3%)	0.387 (60.8%)	0.061 (9.6%)	0.189 (29.6%)	5.1%	3.0%	− 7.5%
Brain	${\dot{D}}_{max}$	0.247 (44.8%)	0.043 (7.8%)	0.262 (47.4%)	0.252 (49.8%)	0.021 (4.2%)	0.233 (46.0%)	2.2%	− 50.2%	− 10.9%
GTV	${\dot{D}}_{max}$	0.557 (66.6%)	0.039 (4.7%)	0.241 (28.8%)	0.559 (65.9%)	0.095 (11.3%)	0.193 (22.8%)	0.4%	143.6%	− 19.6%
	${\dot{D}}_{min}$	0.326 (64.9%)	0.030 (6.0%)	0.146 (29.1%)	0.341 (69.2%)	0.020 (4.1%)	0.132 (26.8%)	4.6%	− 33.3%	− 9.6%

**Figure 5:**
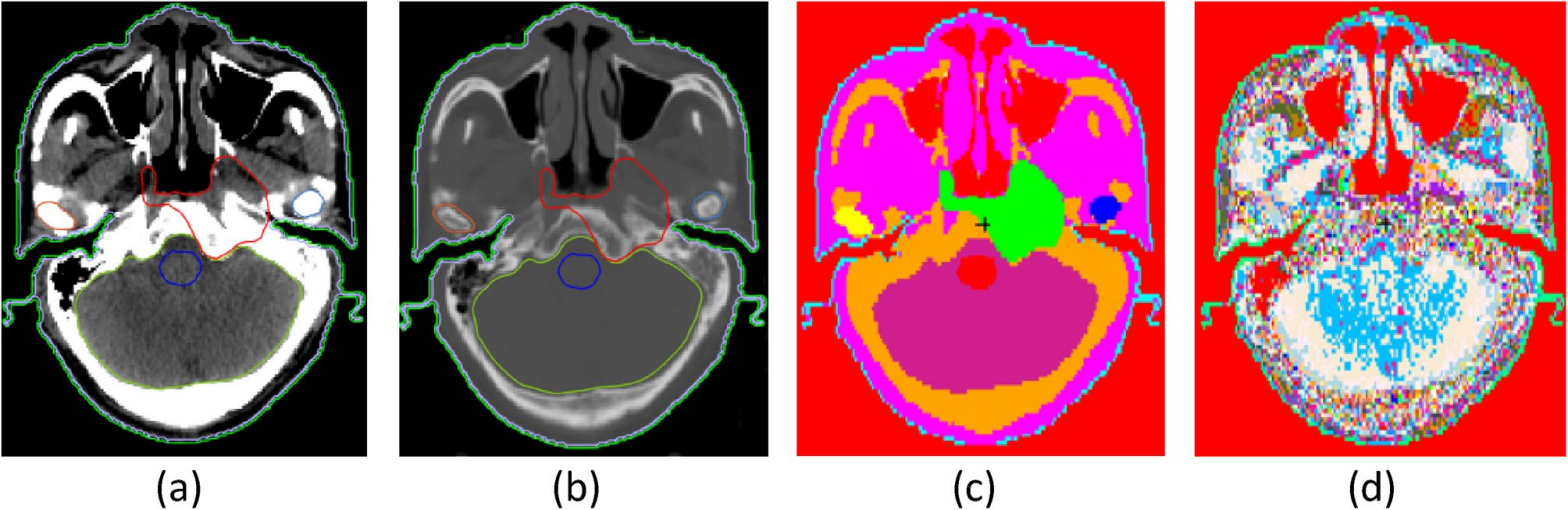
HNC case: (**a**) CT image in the brain window. (**b**) CT image in the bone window. (**c**) Conversion model with specific material from the ICRU-46 report in ROIs. (**d**) Conversion model with multiple materials that were automatically converted to the elemental compositions and densities by the HU values.

The HNC case CT image is shown in the brain window in Fig. 5a and in the bone window in Fig. 5b, and the ROI-GTV (red) invades the soft tissue and skeletal tissue. The conversion model III in Fig. 5c is provided with the average tissue material composition for ROIs as suggested by ICRU, and the conversion model IV in Fig. 5d is provided with the skin tissue material composition for ROI-skin as suggested by ICRU. The rest of the ROIs are provided with multiple materials with the elemental composition and density in Appendix A converted by the HU of images. The HU of GTV in the transformed voxel model has a range of −622 ~ 1557, with the skeletal muscle composition and density of 1.05 g/cm^3^ of ICRU adopted in model III and with the density of 1.1161–1.8145 g/cm^3^ in model IV. The HU of the mandible has a range of 89–1596, and the mandible composition with a density of 1.68 g/cm^3^ from ICRU is adopted in model III, while the density of 1.3257–1.8145 g/cm^3^ is adopted in model IV.

**Figure 6:**
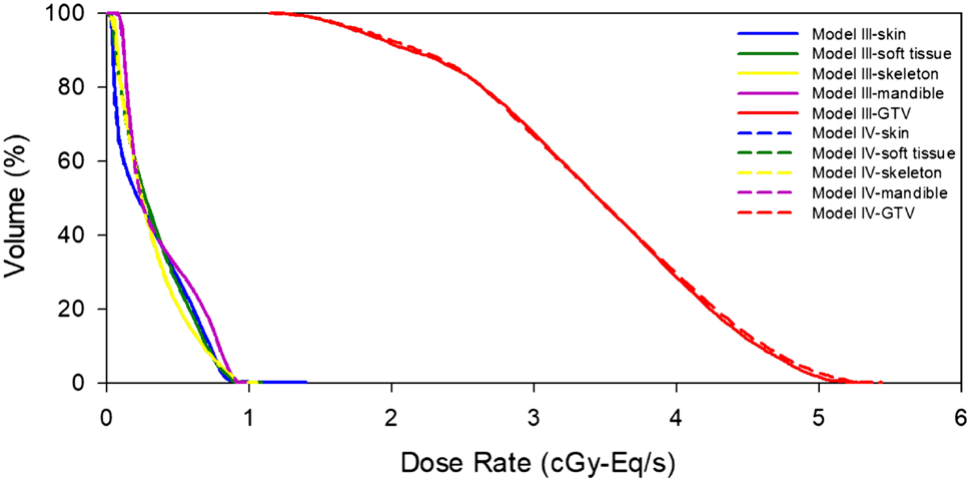
DVHs of the HNC case of conversion models III and IV.

The biologically equivalent dose rate results and DVH of the HNC case are shown in Table 5 and Fig. 6, respectively. The difference of ${\dot{D}}_{max,BE}$ in soft tissue and skeletal tissue between models III and IV was more than 10%. The maximum skeletal tissue biologically equivalent dose rate differed by 6.1% between the two models. The ${\dot{D}}_{mean,BE}$ of GTV to was similar in model III and model IV, but the difference in ${\dot{D}}_{max,BE}$ and ${\dot{D}}_{min,BE}$ was more than 3%. Table 6 shows the biologically equivalent dose component, the proportions in parentheses, and the difference between model III and model IV. The background dose rate of normal tissue accounts for up to 53% of ${\dot{D}}_{max,BE}$, while the corresponding one of GTV accounts for less than 12% of ${\dot{D}}_{max,BE}$ and ${\dot{D}}_{min,BE}$.

**Table 5:** Biologically equivalent dose rate results of HNC case of conversion models III and IV.

ROI	Conversion model III (IntraROI homogeneous)				Conversion model IV (IntraROI heterogeneous)				Difference		
	Mat	${\dot{D}}_{max}$(cGy-Eq/s)	${\dot{D}}_{min}$(cGy-Eq/s)	${\dot{D}}_{mean}$(cGy-Eq/s)	Mat	${\dot{D}}_{max}$(cGy-Eq/s)	${\dot{D}}_{min}$(cGy-Eq/s)	${\dot{D}}_{mean}$(cGy-Eq/s)	${\dot{D}}_{max}$	${\dot{D}}_{min}$	${\dot{D}}_{mean}$
Skin	ICRU_Skin_	1.397 (0.69%)	0.032	0.313 (2.04%)	ICRU_Skin_	1.425 (0.68%)	0.028	0.307 (2.05%)	2.0%	− 12.5%	− 1.7%
Soft tissue	ICRU_ST_	0.941 (0.93%)	0.032	0.335 (1.88%)	HU conv	1.102 (0.85%)	0.029	0.330 (1.89%)	17.1%	− 9.6%	− 1.6%
Skeletal tissue	ICRU_Cranium_	0.966 (0.92%)	0.044	0.315 (1.87%)	HU conv	1.065 (0.87%)	0.042	0.310 (1.89%)	10.2%	− 4.0%	− 1.7%
Mandible	ICRU_Mandible_	0.935 (0.93%)	0.075	0.371 (1.70%)	HU conv	0.992 (0.91%)	0.074	0.367 (1.70%)	6.1%	− 1.8%	− 1.1%
GTV	ICRU_SM_	5.269 (0.64%)	1.182	3.410 (0.79%)	HU conv	5.438 (0.63%)	1.145	3.426 (0.79%)	3.2%	− 3.1%	0.5%

Mat.: Material, ST: Soft Tissue, SM: Skeletal Muscle, HU conv.: HU conversion.

**Table 6:** Biologically equivalent dose rate component of HNC case of conversion models III and IV.

ROI	Dose category	Conversion model III (IntraROI homogeneous)			Conversion model IV (IntraROI heterogeneous)			Difference		
		${\dot{D}}_{B}$(cGy-Eq/s)	${\dot{D}}_{N}$(cGy-Eq/s)	${\dot{D}}_{P}$(cGy-Eq/s)	${\dot{D}}_{B}$(cGy-Eq/s)	${\dot{D}}_{N}$(cGy-Eq/s)	${\dot{D}}_{P}$(cGy-Eq/s)	${\dot{D}}_{B}$	${\dot{D}}_{N}$	${\dot{D}}_{P}$
Skin	${\dot{D}}_{max}$	0.922 (66.0%)	0.235 (16.9%)	0.239 (17.1%)	0.972 (68.2%)	0.241 (16.9%)	0.213 (14.9%)	5.4%	2.3%	− 11.1%
Soft tissue	${\dot{D}}_{max}$	0.498 (52.9%)	0.200 (21.2%)	0.243 (25.8%)	0.524 (47.6%)	0.353 (32.1%)	0.224 (20.3%)	5.2%	76.8%	− 8.1%
Skeletal tissue	${\dot{D}}_{max}$	0.511 (52.6%)	0.212 (21.9%)	0.247 (25.5)	0.530 (56.7%)	0.179 (19.1%)	0.227 (24.2%)	3.7%	− 15.9%	− 8.3%
Mandible	${\dot{D}}_{max}$	0.491 (52.5%)	0.214 (22.9%)	0.230 (24.6%)	0.467 (47.1%)	0.335 (33.7%)	0.190 (19.1%)	− 4.8%	56.5%	− 17.6%
GTV	${\dot{D}}_{max}$	4.851 (92.1%)	0.181 (3.4%)	0.238 (4.5%)	5.004 (92.0%)	0.208 (3.8%)	0.226 (4.2%)	3.2%	15.4%	− 4.9%
	${\dot{D}}_{min}$	1.040 (88.0%)	0.032 (2.7%)	0.109 (9.3%)	1.014 (88.6%)	0.033 (2.9%)	0.097 (8.5%)	− 2.5%	3.0%	− 11.0%

The physically absorbed dose rate results, component, and their differences between model III and model IV are shown in Tables 7 and 8, respectively. The 6.2% difference in the ${\dot{D}}_{max,phy}$ of soft tissue is due mainly to the difference in the ${\dot{D}}_{N,phy}$. The difference in the ${\dot{D}}_{min,phy}$ of GTV was 3.1%. The ${\dot{D}}_{B,phy}$ of model III and model IV account for approximately 70% of the GTV ${\dot{D}}_{min,phy}$, and the difference between the two models is 2.3%. The ${\dot{D}}_{N,phy}$ and ${\dot{D}}_{P,phy}$ of the two models account for approximately 3% and 27%, respectively. Between model III and model IV, ${\dot{D}}_{N,phy}$ and ${\dot{D}}_{P,phy}$ of the GTV ${\dot{D}}_{min,phy}$ differ by 15.7% and 6.7%, respectively. This finding indicates that material composition can have an effect on dose distribution even in tumors with a low background dose.

**Table 7:** Physically absorbed dose rate results of HNC case of conversion models III and IV.

ROI	Conversion model III (IntraROI homogeneous)				Conversion model IV (IntraROI heterogeneous)				Difference		
	Mat	${\dot{D}}_{max}$(cGy/s)	${\dot{D}}_{min}$(cGy/s)	${\dot{D}}_{mean}$(cGy/s)	Mat	${\dot{D}}_{max}$(cGy/s)	${\dot{D}}_{min}$(cGy/s)	${\dot{D}}_{mean}$(cGy/s)	${\dot{D}}_{max}$	${\dot{D}}_{min}$	${\dot{D}}_{mean}$
Skin	ICRU_Skin_	0.683 (0.74%)	0.029	0.156 (2.20%)	ICRU_Skin_	0.681 (0.71%)	0.026	0.150 (2.19%)	− 0.3%	− 11.3%	− 3.7%
Soft tissue	ICRU_ST_	0.699 (0.74%)	0.031	0.241 (1.65%)	HU conv	0.742 (0.67%)	0.029	0.234 (163%)	6.2%	− 5.6%	− 2.8%
Skeletal tissue	ICRU_Cranium_	0.702 (0.71%)	0.040	0.233 (1.62%)	HU conv	0.714 (0.68%)	0.038	0.227 (1.59%)	1.7%	− 4.7%	− 2.7%
Mandible	ICRU_Mandible_	0.675 (0.72%)	0.065	0.271 (1.40%)	HU conv	0.680 (0.71%)	0.063	0.267 (1.37%)	0.8%	− 2.3%	− 1.2%
GTV	ICRU_SM_	1.576 (0.66%)	0.393	1.048 (0.81%)	HU conv	1.610 (0.65%)	0.381	1.048 (0.80%)	2.1%	− 3.1%	0.0%

Mat.: Material, ST: Soft Tissue, SM: Skeletal Muscle, HU conv.: HU conversion.

**Table 8:** Physically absorbed dose rate component of HNC case of conversion models III and IV.

ROI	Dose Category	Conversion model III (IntraROI homogeneous)			Conversion model IV (IntraROI heterogeneous)			Difference		
		${\dot{D}}_{B}$(cGy/s)	${\dot{D}}_{N}$(cGy/s)	${\dot{D}}_{P}$(cGy/s)	${\dot{D}}_{B}$(cGy/s)	${\dot{D}}_{N}$(cGy/s)	${\dot{D}}_{P}$(cGy/s)	${\dot{D}}_{B}$	${\dot{D}}_{N}$	${\dot{D}}_{P}$
Skin	${\dot{D}}_{max}$	0.366 (53.5%)	0.072 (10.6%)	0.245 (35.9%)	0.381 (58.3%)	0.048 (7.3%)	0.225 (34.4%)	4.2%	− 34.0%	− 8.3%
Soft Tissue	${\dot{D}}_{max}$	0.386 (55.2%)	0.051 (7.3%)	0.262 (37.5%)	0.409 (55.1%)	0.106 (14.3%)	0.227 (30.6%)	6.0%	106.9%	− 13.3%
Skeletal Tissue	${\dot{D}}_{max}$	0.393 (56.0%)	0.066 (9.5%)	0.243 (34.6%)	0.387 (54.3%)	0.098 (13.7%)	0.228 (32.0%)	− 1.4%	47.4%	− 5.9%
Mandible	${\dot{D}}_{max}$	0.378 (56.0%)	0.067 (9.9%)	0.230 (34.1%)	0.395 (58.1%)	0.065 (9.6%)	0.220 (32.4%)	4.6%	− 2.8%	− 4.5%
GTV	${\dot{D}}_{max}$	1.272 (80.7%)	0.055 (3.5%)	0.248 (15.8%)	1.318 (81.9%)	0.065 (4.0%)	0.226 (14.1%)	3.6%	17.8%	− 8.9%
	${\dot{D}}_{min}$	0.274 (69.8%)	0.010 (2.6%)	0.109 (27.6%)	0.268 (70.3%)	0.012 (3.1%)	0.101 (26.6%)	− 2.3%	15.7%	− 6.7%

## Discussion and conclusion

The differences between the intraROI homogeneous and heterogeneous models are pronounced and prominent, which is ranged from a few to more than ten percent as found in conversion models I to IV. The change in material composition (especially the ratio of hydrogen atoms) attributes to the change in neutron moderation as well as other nuclear reactions, which leads to a change in neutron energy as well as spatial distributions within the body. As a result, the estimated doses are different between the two conversion models. It is clear that some anatomy structural information was erased when using the conventional conversion method, while the proposed new method could preserve as much information as possible and provides more details in constructing the following voxel phantom used in the Monte Carlo dose engine. That is to say, the conventional material conversion model used in most BNCT TPSs has significant defects which could not reflect faithfully the real material composition of the human body as well as OARs; this causes a deviation in dose estimation and delivers an inaccurate dose plan. Thus, one should use the HU-based conversion model, demonstrated in this work with 96 corresponding materials, in determining the tissue material composition as well as the density of each pixel read from CT images. However, one tissue has a range of corresponding HU values, and the HU values overlap with other tissues. Although the use of HU-value conversion for ROI material determination has a significant advantage in reflecting the variety of material compositions and densities, its application requires additional care with the input of anatomical knowledge and other aids. There is still room for the material conversion study, and the next TPS version will propose a more dedicated, updated method to achieve a conversion that more closely matches the actual physiological and pathological conditions.

### Supplementary Information

Supplementary Information.

## Data Availability

The datasets used and/or analyzed during the current study are available from the corresponding author upon reasonable request.
