# Supplementary material for: HU-based material conversion for BNCT accurate dose estimation
Source: Sci Rep. 2023 Sep 21;13:15701. doi: 10.1038/s41598-023-42508-0 (PMC10514297; doi:10.1038/s41598-023-42508-0)
Supplement: Supplementary file 1 — Supplementary Information. [file 41598_2023_42508_MOESM1_ESM.docx]

# **Supplementary information**

**Appendix A: HU values correspond to 96 kinds of tissue densities and elemental compositions**

| **HU_L_** | **HU_U_** | **ρ**  **(g/cm^3^)** | **H** | **C** | **N** | **O** | **P** | **Ca** | **Ar** | **HU_L_** | **HU_U_** | **ρ**  **(g/cm^3^)** | **H** | **C** | **N** | **O** | **P** | **Ca** |
| --- | --- | --- | --- | --- | --- | --- | --- | --- | --- | --- | --- | --- | --- | --- | --- | --- | --- | --- |
| min | -990 | 1.29E-03 | 0 | 0 | 75.5 | 23.2 | 0 | 0 | 1.3 | 325 | 350 | 1.4071 | 6.3 | 26.4 | 3.9 | 43.9 | 6.1 | 13.3 |
| -990 | -980 | 0.9300 | 11.6 | 68.3 | 0.2 | 19.9 | 0.0 | 0.0 | 0 | 350 | 375 | 1.4186 | 6.3 | 32.0 | 3.1 | 37.9 | 6.5 | 14.2 |
| -980 | -970 | 0.9500 | 11.4 | 60.0 | 0.7 | 27.9 | 0.0 | 0.0 | 0 | 375 | 400 | 1.4300 | 6.3 | 32.7 | 3.0 | 36.9 | 6.6 | 14.5 |
| -970 | -960 | 0.9700 | 11.2 | 51.9 | 1.3 | 35.6 | 0.0 | 0.0 | 0 | 400 | 425 | 1.4420 | 6.1 | 28.7 | 3.5 | 40.6 | 6.6 | 14.4 |
| -960 | -950 | 0.9800 | 11.5 | 64.6 | 0.7 | 23.2 | 0.0 | 0.0 | 0 | 425 | 450 | 1.4540 | 6.0 | 26.4 | 3.7 | 42.5 | 6.7 | 14.6 |
| -950 | -925 | 0.9900 | 10.9 | 50.8 | 2.3 | 35.9 | 0.1 | 0.0 | 0 | 450 | 475 | 1.4600 | 6.0 | 30.9 | 3.2 | 37.7 | 7.0 | 15.2 |
| -925 | -900 | 1.0000 | 11.0 | 53.1 | 2.1 | 33.6 | 0.1 | 0.0 | 0 | 475 | 500 | 1.4660 | 6.0 | 31.2 | 3.1 | 37.1 | 7.0 | 15.4 |
| -900 | -875 | 1.0200 | 10.7 | 33.2 | 3.0 | 52.9 | 0.2 | 0.0 | 0 | 500 | 525 | 1.4780 | 5.9 | 30.8 | 3.2 | 37.4 | 7.1 | 15.7 |
| -875 | -850 | 1.0271 | 11.7 | 31.5 | 4.2 | 52.4 | 0.2 | 0.0 | 0 | 525 | 550 | 1.4900 | 5.8 | 30.3 | 3.2 | 37.6 | 7.2 | 15.9 |
| -850 | -825 | 1.0414 | 10.8 | 7.3 | 1.1 | 80.8 | 0.0 | 0.0 | 0 | 550 | 575 | 1.5050 | 5.7 | 26.9 | 3.6 | 40.7 | 7.3 | 15.8 |
| -825 | -800 | 1.0557 | 10.3 | 14.0 | 3.2 | 72.2 | 0.2 | 0.0 | 0 | 575 | 600 | 1.5200 | 5.6 | 23.7 | 4.0 | 43.7 | 7.3 | 15.7 |
| -800 | -775 | 1.0700 | 10.2 | 12.7 | 3.3 | 73.4 | 0.3 | 0.0 | 0 | 600 | 625 | 1.5260 | 5.6 | 23.5 | 4.0 | 43.7 | 7.4 | 15.8 |
| -775 | -750 | 1.0775 | 10.1 | 20.6 | 4.2 | 65.0 | 0.1 | 0.0 | 0 | 625 | 650 | 1.5380 | 5.5 | 23.2 | 4.0 | 43.7 | 7.5 | 16.1 |
| -750 | -725 | 1.0925 | 9.5 | 21.0 | 6.3 | 63.1 | 0.0 | 0.0 | 0 | 650 | 675 | 1.5500 | 5.4 | 22.9 | 4.0 | 43.7 | 7.6 | 16.4 |
| -725 | -700 | 1.1000 | 9.8 | 10.1 | 2.2 | 75.7 | 2.2 | 0.0 | 0 | 675 | 700 | 1.5620 | 5.3 | 22.6 | 4.0 | 43.7 | 7.7 | 16.6 |
| -700 | -650 | 1.1054 | 9.9 | 10.9 | 2.3 | 74.5 | 2.2 | 0.2 | 0 | 700 | 725 | 1.5740 | 5.2 | 22.3 | 4.0 | 43.8 | 7.8 | 16.9 |
| -650 | -600 | 1.1161 | 9.8 | 12.5 | 2.4 | 72.2 | 2.3 | 0.9 | 0 | 725 | 750 | 1.5860 | 5.2 | 22.0 | 4.0 | 43.8 | 7.9 | 17.1 |
| -600 | -550 | 1.1268 | 9.7 | 14.1 | 2.5 | 69.8 | 2.5 | 1.4 | 0 | 750 | 775 | 1.5980 | 5.1 | 21.7 | 4.0 | 43.8 | 8.0 | 17.4 |
| -550 | -500 | 1.1375 | 9.6 | 15.7 | 2.6 | 67.4 | 2.6 | 2.0 | 0 | 775 | 800 | 1.6100 | 5.0 | 21.4 | 4.0 | 43.8 | 8.1 | 17.6 |
| -500 | -450 | 1.1482 | 9.9 | 17.3 | 2.7 | 65.1 | 2.7 | 2.2 | 0 | 800 | 825 | 1.6158 | 5.0 | 21.2 | 4.0 | 43.8 | 8.1 | 17.8 |
| -450 | -400 | 1.1589 | 9.0 | 19.8 | 2.9 | 62.7 | 2.9 | 2.7 | 0 | 825 | 850 | 1.6275 | 4.9 | 21.0 | 4.0 | 43.8 | 8.2 | 17.9 |
| -400 | -350 | 1.1696 | 9.0 | 20.9 | 2.8 | 61.4 | 2.9 | 3.0 | 0 | 850 | 875 | 1.6392 | 4.8 | 20.8 | 4.0 | 43.8 | 8.3 | 18.1 |
| -350 | -300 | 1.1804 | 8.8 | 22.1 | 3.1 | 59.0 | 3.0 | 4.0 | 0 | 875 | 900 | 1.6508 | 4.8 | 20.6 | 4.1 | 43.8 | 8.4 | 18.3 |
| -300 | -250 | 1.1911 | 8.6 | 23.7 | 3.2 | 56.7 | 3.2 | 4.5 | 0 | 900 | 925 | 1.6625 | 4.7 | 20.3 | 4.1 | 43.8 | 8.5 | 18.5 |
| -250 | -200 | 1.2018 | 8.5 | 25.3 | 3.3 | 53.9 | 3.3 | 5.6 | 0 | 925 | 950 | 1.6742 | 4.6 | 20.1 | 4.1 | 43.8 | 8.7 | 18.7 |
| -200 | -175 | 1.2125 | 8.3 | 26.9 | 3.5 | 50.9 | 3.5 | 6.3 | 0 | 950 | 975 | 1.6800 | 4.6 | 20.0 | 4.1 | 43.8 | 8.7 | 18.8 |
| -175 | -150 | 1.2232 | 8.2 | 28.5 | 3.6 | 48.6 | 3.6 | 6.9 | 0 | 975 | 1000 | 1.6858 | 4.6 | 20.0 | 4.1 | 43.6 | 8.8 | 18.9 |
| -150 | -125 | 1.2339 | 8.0 | 30.1 | 3.7 | 46.2 | 3.7 | 7.5 | 0 | 1000 | 1050 | 1.6975 | 4.5 | 20.1 | 4.0 | 43.3 | 8.9 | 19.2 |
| -125 | -100 | 1.2446 | 7.9 | 31.7 | 3.8 | 44.0 | 3.8 | 8.1 | 0 | 1050 | 1100 | 1.7092 | 4.4 | 20.2 | 4.0 | 43.0 | 9.0 | 19.4 |
| -100 | -80 | 1.2500 | 7.8 | 31.8 | 3.7 | 44.1 | 4 | 8.6 | 0 | 1100 | 1150 | 1.7208 | 4.4 | 20.3 | 3.9 | 42.7 | 9.1 | 19.6 |
| -80 | -60 | 1.2557 | 7.7 | 31.6 | 3.7 | 44.1 | 4.1 | 8.8 | 0 | 1150 | 1200 | 1.7325 | 4.3 | 20.4 | 3.9 | 42.4 | 9.2 | 19.9 |
| -60 | -40 | 1.2671 | 7.6 | 31.2 | 3.7 | 44.1 | 4.2 | 9.2 | 0 | 1200 | 1250 | 1.7442 | 4.2 | 20.4 | 3.8 | 42.0 | 9.3 | 20.1 |
| -40 | -20 | 1.2786 | 7.5 | 30.8 | 3.7 | 44.1 | 4.4 | 9.5 | 0 | 1250 | 1300 | 1.7500 | 4.2 | 20.5 | 3.8 | 41.9 | 9.4 | 20.2 |
| -20 | 0 | 1.2900 | 7.4 | 30.1 | 3.7 | 44.4 | 4.5 | 9.9 | 0 | 1300 | 1350 | 1.7559 | 4.2 | 20.4 | 3.8 | 41.8 | 9.4 | 20.3 |
| 0 | 20 | 1.3000 | 7.4 | 27.0 | 3.6 | 47.3 | 4.8 | 9.9 | 0 | 1350 | 1400 | 1.7676 | 4.1 | 20.1 | 3.8 | 42.0 | 9.5 | 20.5 |
| 20 | 40 | 1.3043 | 7.4 | 26.6 | 3.6 | 47.6 | 4.9 | 10.0 | 0 | 1400 | 1450 | 1.7793 | 4.1 | 19.7 | 3.9 | 42.1 | 9.6 | 20.7 |
| 40 | 60 | 1.3129 | 7.3 | 26.3 | 3.6 | 47.5 | 5.0 | 10.3 | 0 | 1450 | 1500 | 1.7910 | 4.0 | 19.4 | 3.9 | 42.3 | 9.6 | 20.8 |
| 60 | 80 | 1.3214 | 7.2 | 26.2 | 3.6 | 47.2 | 5.1 | 10.6 | 0 | 1500 | 1550 | 1.8028 | 4.0 | 19.0 | 3.9 | 42.4 | 9.7 | 21.0 |
| 80 | 100 | 1.3257 | 7.0 | 28.6 | 3.8 | 44.3 | 5.1 | 11.1 | 0 | 1550 | 1600 | 1.8145 | 3.9 | 18.7 | 3.9 | 42.5 | 9.8 | 21.1 |
| 100 | 125 | 1.3300 | 7.1 | 35.7 | 2.9 | 36.8 | 5.5 | 11.9 | 0 | 1600 | 1650 | 1.8262 | 3.8 | 18.3 | 4.0 | 42.7 | 9.8 | 21.3 |
| 125 | 150 | 1.3360 | 7.1 | 37.9 | 2.6 | 34.4 | 5.6 | 12.3 | 0 | 1650 | 1700 | 1.8379 | 3.8 | 18.0 | 4.0 | 42.8 | 9.9 | 21.5 |
| 150 | 175 | 1.3480 | 7.0 | 37.3 | 2.7 | 34.6 | 5.8 | 12.6 | 0 | 1700 | 1750 | 1.8497 | 3.7 | 17.7 | 4.0 | 43.0 | 10.0 | 21.6 |
| 175 | 200 | 1.3600 | 6.9 | 36.7 | 2.7 | 34.8 | 5.9 | 12.9 | 0 | 1750 | 1800 | 1.8614 | 3.7 | 17.3 | 4.1 | 43.1 | 10.0 | 21.8 |
| 200 | 225 | 1.3800 | 6.7 | 28.4 | 3.4 | 43.3 | 5.8 | 12.3 | 0 | 1800 | 1850 | 1.8731 | 3.6 | 17.0 | 4.1 | 43.2 | 10.1 | 22.0 |
| 225 | 250 | 1.3825 | 6.6 | 25.2 | 3.7 | 46.4 | 5.7 | 12.2 | 0 | 1850 | 1900 | 1.8848 | 3.6 | 16.6 | 4.1 | 43.4 | 10.2 | 22.1 |
| 250 | 275 | 1.3875 | 6.6 | 29.3 | 3.5 | 41.7 | 5.9 | 12.9 | 0 | 1900 | 1950 | 1.8966 | 3.5 | 16.3 | 4.1 | 43.5 | 10.3 | 22.3 |
| 275 | 300 | 1.3900 | 6.7 | 34.6 | 2.9 | 36.0 | 6.2 | 13.5 | 0 | 1950 | 2000 | 1.9083 | 3.5 | 15.9 | 4.2 | 43.7 | 10.3 | 22.4 |
| 300 | 325 | 1.3957 | 6.6 | 31.6 | 3.3 | 38.9 | 6.1 | 13.4 | 0 | 2000 | max | 1.9200 | 3.4 | 15.6 | 4.2 | 43.8 | 10.4 | 22.6 |

* HU_L_ is the lower limit of the image HU value; HU_U_ is the upper limit of the image HU value.
